# Construction of a Nomogram to Predict Overall Survival in Patients with Early-Onset Hepatocellular Carcinoma: A Retrospective Cohort Study

**DOI:** 10.3390/cancers15225310

**Published:** 2023-11-07

**Authors:** Tianrui Kuang, Wangbin Ma, Jiacheng Zhang, Jia Yu, Wenhong Deng, Keshuai Dong, Weixing Wang

**Affiliations:** 1 Department of General Surgery, Renmin Hospital of Wuhan University, Wuhan 430060, China; 2 Central Laboratory, Renmin Hospital of Wuhan University, Wuhan 430060, China

**Keywords:** nomogram, early-onset hepatocellular carcinoma, overall survival, SEER database, online application

## Abstract

**Simple Summary:**

Hepatocellular carcinoma (HCC) is a severe global health concern, and it is increasingly jeopardizing younger individuals. Despite this, there is a lack of available tools for the prognosis estimation of early-onset HCC. In our study, we conducted a retrospective analysis of early-onset HCC (EO-LIHC) using data of the period from 2004 to 2018. We identified independent risk factors using a Cox regression analysis, including age, sex, AFP level, the grading and staging of the tumor, the size of the tumor, and whether the patient was receiving therapy like surgery and chemotherapy. We developed a predictive nomogram to estimate 1-, 3-, and 5-year survival rates of EO-LIHC patients and a user-friendly web-based survival prediction model tailored for these patients. These findings provide valuable insights for personalized care and treatment decisions for individuals with EO-LIHC.

**Abstract:**

Hepatocellular carcinoma (HCC) is a widespread and impactful cancer which has pertinent implications worldwide. Although most cases of HCC are typically diagnosed in individuals aged ≥60 years, there has been a notable rise in the occurrence of HCC among younger patients. However, there is a scarcity of precise prognostic models available for predicting outcomes in these younger patients. A retrospective analysis was conducted to investigate early-onset hepatocellular carcinoma (EO-LIHC) using data from the Surveillance, Epidemiology, and End Results (SEER) database from 2004 to 2018. The analysis included 1392 patients from the SEER database and our hospital. Among them, 1287 patients from the SEER database were assigned to the training cohort (*n* = 899) and validation cohort 1 (*n* = 388), while 105 patients from our hospital were assigned to validation cohort 2. A Cox regression analysis showed that age, sex, AFP, grade, stage, tumor size, surgery, and chemotherapy were independent risk factors. The nomogram developed in this study demonstrated its discriminatory ability to predict the 1-, 3-, and 5-year overall survival (OS) rates in EO-LIHC patients based on individual characteristics. Additionally, a web-based OS prediction model specifically tailored for EO-LIHC patients was created and validated. Overall, these advancements contribute to improved decision-making and personalized care for individuals with EO-LIHC.

## 1. Introduction

Cancer is a complex disease influenced by multiple factors, primarily impacting individuals aged ≥50 years. Early-onset malignancies, which are those detected in persons aged <50 years, have become more common in several nations in recent decades. This upward trend is particularly evident in colorectal, gallbladder, liver, pancreatic, and other gastrointestinal tumors [1,2,3,4]. Although the overall incidence and mortality rates of all types of cancer are significantly lower in younger adults compared to older adults, cancers in younger men and women have substantial economic and social implications. Moreover, they can lead to a higher loss of person-years of life compared to cancers diagnosed later in life [5,6,7].

Hepatocellular carcinoma (HCC) is recognized as one of the most common cancers worldwide, presenting a significant healthcare challenge on a global scale [8]. Although most liver cancer cases are diagnosed in individuals aged ≥60 years, a consistent increase in the incidence of liver cancer has been reported among young patients recently. Several risk factors for HCC are now clearly defined, such as chronic hepatitis B and C virus infections, chronic hepatitis, cirrhosis, and alcohol consumption [9]. However, limited information is available regarding risk factors specifically associated with early-onset hepatocellular carcinoma (EO-LIHC), which warrants further investigation and understanding. It has been shown that patients with EO-LIHC not only have fewer complications but also have a lower incidence of underlying cirrhosis compared to patients with advanced HCC, suggesting that there may be an underlying etiologic difference between the two groups of patients [10]. Distinctions in epidemiology, clinical manifestations, pathological characteristics, and molecular features between early-stage and late-stage cancers have been observed. However, it is unlikely to precisely demarcate these disparities at the age of 50 [11].

Current clinical practice recommendations heavily rely on the AJCC TNM staging system to predict prognosis and guide therapeutic decisions for patients with EO-LIHC [12]. However, the TNM staging system has some limitations due to various factors that can influence patient prognosis, including age, gender, the degree of tumor differentiation, serum biomarkers, and treatment-related variables. Nomograms have shown precise predictive capabilities for various tumor types and are widely used in clinical settings, surpassing the traditional TNM staging system or alternative staging systems [13,14,15]. However, currently, there is a lack of a nomogram model specifically developed to predict postoperative survival in patients with EO-LIHC.

In this context, we constructed and validated a novel nomogram for the first time, utilizing data from the SEER database and a Chinese cohort. With the use of this nomogram, patients with EO-LIHC can have their overall survival (OS) predicted. Additionally, recognizing the significance of improving medical care for patients, we developed a web-based model. This web-based tool enables clinicians to make more informed clinical decisions by accurately assessing the prognosis of patients with EO-LIHC.

## 2. Methods

### 2.1. Study Design and Cohort Selection

The precise definition of EO-LIHC remains controversial, particularly regarding the age cutoff. For this study, EO-LIHC was defined as HCC diagnosis in patients aged ≤50 years. The study cohorts were established by screening patients diagnosed with HCC between 2004 and 2018 using the SEER database. From the SEER database, 1287 EO-LIHC patients were randomly assigned to the training cohort (*n* = 899) and validation cohort 1 (*n* = 388) in a 7:3 ratio. Furthermore, 105 EO-LIHC patients from our hospital comprised validation cohort 2.

**Figure 1:**
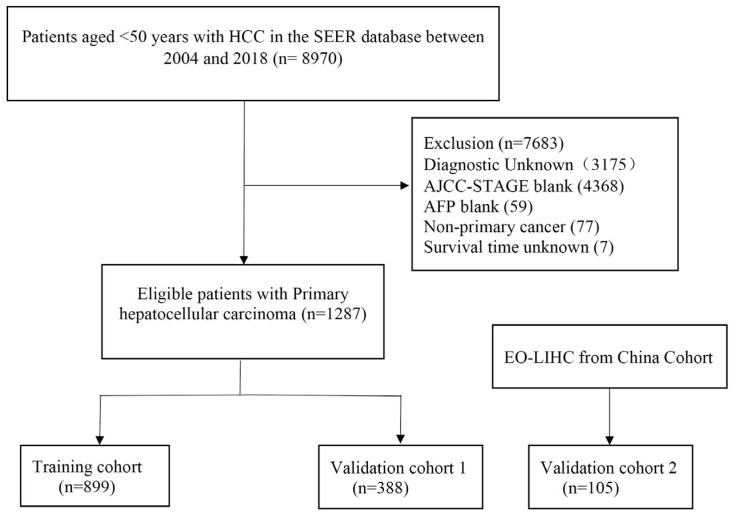
Flow diagram of selecting patients.

Specific inclusion and exclusion criteria were applied to all the cohorts. The inclusion criteria were as follows: (a) age < 50 years; (b) confirmed diagnosis of HCC; and (c) known cause of death. The exclusion criteria were as follows: (a) lack of clinicopathological information; (b) lack of follow-up information; and (c) missing treatment options. Figure 1 shows the detailed information regarding the inclusion and exclusion of the patients. This retrospective study has been reported in line with the Strengthening the Reporting of Cohort Studies in Surgery guidelines [16].

### 2.2. Variables Management

We retrieved 11 clinically relevant variables for EO-LIHC from the SEER database, which included data regarding age, sex, race, pathology, tumor size, AJCC TNM stage, alpha-fetoprotein (AFP) levels, chemotherapy, and survival. The primary outcome of the research was OS, which was calculated as the period until death. The tumors were staged according to the AJCC TNM staging criteria, the 8th edition.

### 2.3. Establishment and Validation of the Nomogram Model

A prognostic model was constructed using a nomogram with the training cohort. The stability of the model was assessed using validation cohorts 1 and 2. To determine the factors that substantially affect OS in patients with EO-LIHC, univariate and multivariate Cox regression analyses were conducted on all variables included in the research.

The models were verified based on the training and validation cohorts using the C-index, receiver-operating characteristic (ROC) curve, calibration curves, and decision curve analysis (DCA). The C-index was used to examine the nomogram’s performance and prediction accuracy, while the ROC curve was used to evaluate its sensitivity and specificity. Calibration curves were generated for 1-, 3-, and 5-year periods to assess the agreement between the model predictions and actual data.

### 2.4. Comparison of Nomogram-Based Risk Classification and the AJCC TNM Staging System

To evaluate the comparative net benefit and risk stratification of the nomogram model against the AJCC TNM staging system, several statistical measures were employed, including the net reclassification index (NRI), C-index, integrated discrimination improvement (IDI), and decision curve analysis (DCA). The DCA was specifically utilized to gauge the clinical utility of the nomogram. To facilitate risk stratification, all eligible patients were categorized into low-risk, middle-risk, and high-risk groups. The optimal threshold for determining the overall score was determined using an X-Tile analysis. The OS of patients in the various risk categories was then compared using Kaplan–Meier curves and log-rank testing.

### 2.5. Statistical Methods

Descriptive statistics for all study variables are presented as numbers and percentages. To explore the associations between variables and survival outcomes, both univariate and multivariable Cox regression analyses were performed using R, version 3.6.3, and relevant packages for the correlation analysis. Various evaluation metrics, such as the C-index and ROC curve, were used to assess the performance of the model. OS was analyzed using Kaplan–Meier and log-rank tests. The chi-square test was used to determine whether the distributions of the cohorts used for training and validation differed. Two-tailed *p*-values < 0.05 were considered statistically significant.

## 3. Results

### 3.1. Patient Characteristics

Patients confirmed with HCC between 2004 and 2018 were identified using the SEER database. In total, 1287 patients with EO-LIHC from the database were randomly divided into a training cohort (*n* = 899) and validation cohort 1 (*n* = 388) in a 7:3 ratio. Furthermore, validation cohort 2 comprised 105 EO-LIHC patients from our hospital. In the study sample (*n* = 1392), 985 patients were male, 452 had tumors measuring <5 cm, 690 tested positive for AFP, and 485 received chemotherapy. No significant differences were observed between the training cohort and validation cohort 1 in the patient characteristics (Table 1). Supplementary Table S1 shows the details of the patients in validation cohort 2.

### 3.2. Univariate and Multivariate Analyses

In our endeavor to comprehensively assess the influence of each prognostic factor on the ultimate outcome, we meticulously conducted both univariate and multivariate Cox regression analyses within the training cohort. The results of the univariate analysis notably unveiled statistically significant differences in the training cohort across several key variables, including age, gender, tumor stage, alpha-fetoprotein (AFP) level, tumor grade, tumor size, surgical procedures, the extent of lymph node removal, and even marital status, as thoughtfully summarized in Table 2.

**Figure 2:**
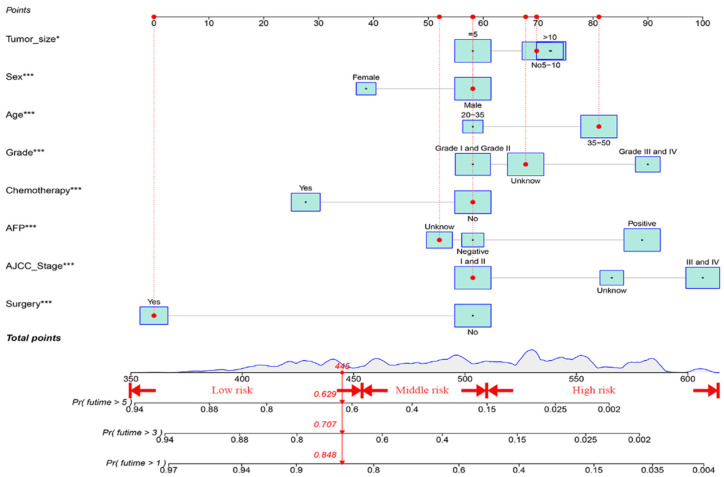
The nomogram predicts the 1-, 3-, and 5- Years OS rates in HCC patients with EO-LIHC. Significant difference: * *p*  <  0.05, *** *p*  <  0.001.

Subsequently, employing a rigorous multiple regression analysis approach, we further distilled our findings to identify the truly independent prognostic factors for overall survival (OS). This analysis underscored the pivotal role of age, gender, AFP levels, tumor grade, tumor stage, tumor size, surgical interventions, and the judicious use of chemotherapy as determinants of OS. These critical factors were seamlessly incorporated into our comprehensive nomogram, as visually depicted in Figure 2. Our work serves as a testament to the vital role these factors play in the precise prediction of OS within our studied cohort.

### 3.3. Construction and Verification of the OS Prognostic Nomogram

Figure 2 presents the prediction results of the nomogram constructed to estimate the 1-, 3-, and 5-year OS rates of the EO-LIHC patients. The nomogram integrated all independent prognostic markers determined by the multivariate Cox regression model. The nomogram offers a personalized approach for prognostication by considering various clinicopathological characteristics of individual patients.

**Figure 3:**
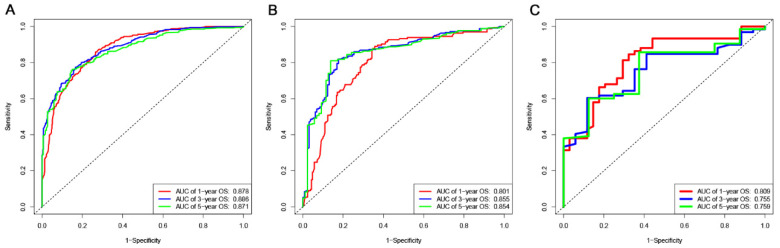
The ROC curves of the nomogram to predict 1-, 3-, and 5-year OS: (**A**) the training cohort; (**B**) the validation cohort 1; and (**C**) the validation cohort 2.

**Figure 4:**
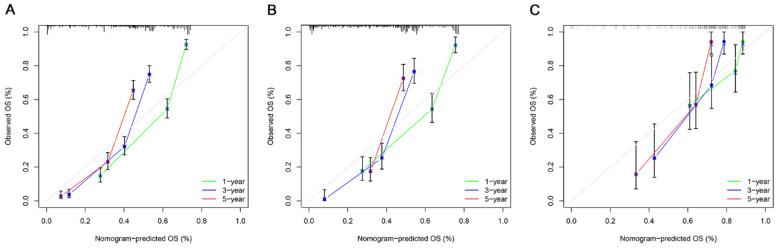
The calibration curves of the nomograms using three cohorts show how survival predictions from the model compare to the actual observed survival; the calibration curve of 1-, 3-, and 5-year OS: (**A**) the training cohort; (**B**) the validation cohort 1; and (**C**) the validation cohort 2.

**Figure 5:**
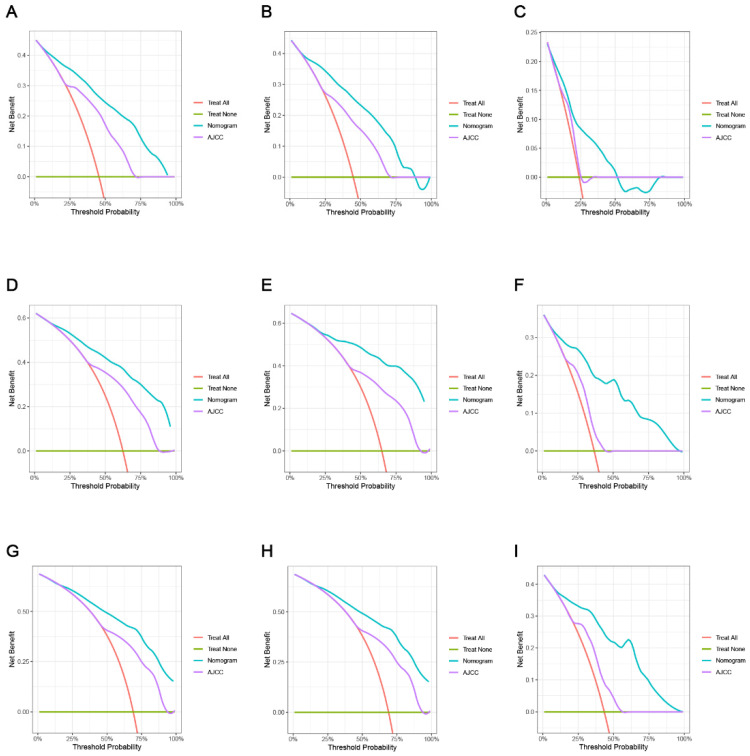
Decision curve analysis of the nomogram and AJCC tumor staging for the overall survival prediction of HCC patients with EO-LIHC. (**A**,**D**,**G**) 1-, 3- and 5-year overall survival benefits based on the training cohorts. (**B**,**E**,**H**) 1-, 3- and 5-year overall survival benefits based on the training cohort 1. (**C**,**F**,**I**) 1-, 3- and 5-year overall survival benefits based on the training cohort 2.

**Figure 6:**
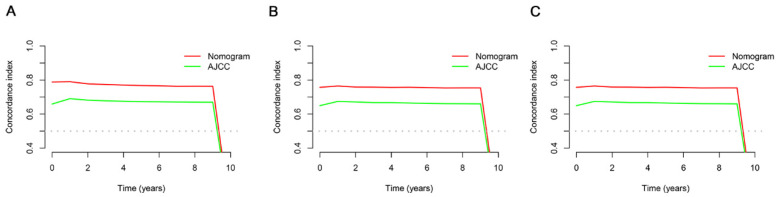
The comparison chart of C-index between nomogram and AJCC stage: (**A**) the training cohort; (**B**) the validation cohort 1; and (**C**) the validation cohort 2.

The ROC, DCA, and calibration curves were generated to complement this analysis (Figure 3, Figure 4 and Figure 5). The internal validation was performed using the C-index to evaluate the model’s correctness and showed the following values: training cohort, 0.784 (95% confidence interval [CI]: 0.777–0.797); validation cohort 1, 0.781 (95% CI: 0.763–0.804); and validation cohort 2, 0.766 (95% CI: 0.731–0.782) (Figure 6). The area under the ROC curve values were 0.878, 0.886, and 0.871 for the training cohort at the 1-, 3-, and 5-year time points, respectively. This indicates the robust predictive performance of the nomogram across these intervals. Additionally, the calibration curves exhibited strong concordance between the predicted and observed probabilities of the 1-, 3-, and 5-year OS rates. Furthermore, the DCA curves revealed favorable clinical net benefits at 1-, 3-, and 5-year intervals for the training cohort as well as the validation cohorts 1 and 2, underscoring the utility of the nomogram in guiding clinical decision-making.

### 3.4. Comparing the Clinical Applicability of the New Nomogram with the AJCC TNM Staging System

We used a thorough study based on the NRI, IDI, and C-index to compare the relative benefits and drawbacks of the nomogram with the conventional AJCC TNM staging system. Our investigation yielded noteworthy findings, illustrating that the nomogram outperformed the AJCC staging system, as evidenced by the higher C-index values in the former than in the latter (Figure 6). The IDI values for 1-, 3-, and 5-year OS in the training cohort were 0.197, 0.204, and 0.189, respectively, while those in validation cohort 1 were 0.228, 0.306, and 0.323, respectively (*p* < 0.05). These findings conclusively prove that the established nomogram is better than the AJCC TNM staging system. Furthermore, the NRI values in the training cohort at 1 year, 3 years, and 5 years were 0.794 (95% CI: 0.636–0.929), 0.699 (95% CI: 0.211–0.814), and 0.204 (95% CI: 0.099–0.328), respectively (Table 3). Additionally, we compared the net benefit of the nomogram and the AJCC TNM staging system using the DCA to assess their practical benefits. Notably, the DCA curves consistently demonstrated that the nomogram provided superior predictions for the 1-, 3-, and 5-year OS rates across all cohorts, delivering greater net benefits when compared to the AJCC TNM staging system.

### 3.5. Establishment of a Risk Stratification System According to the Nomogram

**Figure 7:**
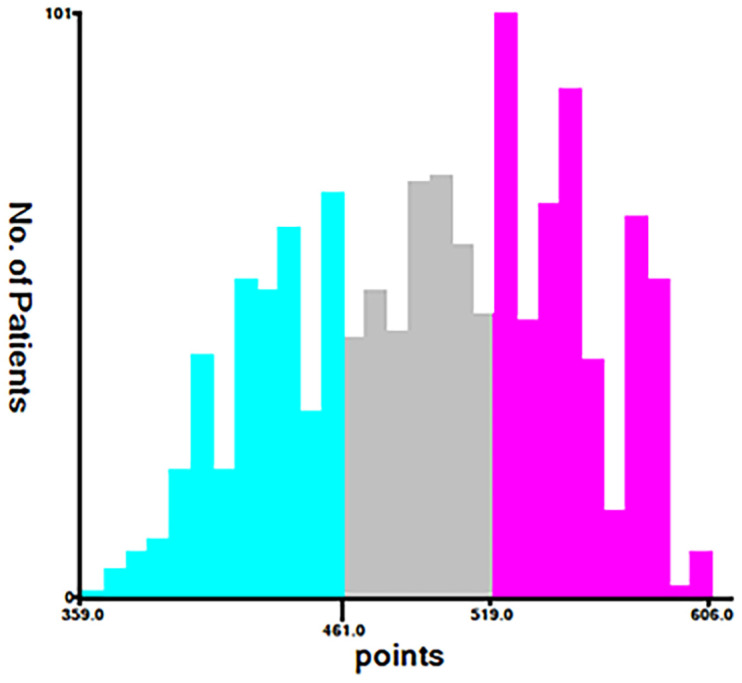
The basis for the grouping new risk stratification (cut-off point selected using the X-tile). blue part (low risk: total score < 461), gray part (middle risk: 461 ≤ total score < 519), and purple part (high risk: total score ≥ 519).

**Figure 8:**
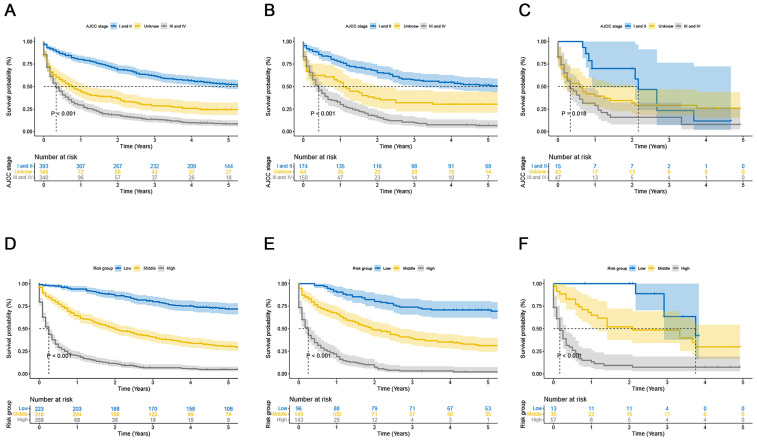
Kaplan–Meier curves of overall survival for the new risk classification and the AJCC tumor staging: (**A**) the AJCC tumor staging in the training cohort; (**B**) the AJCC tumor staging in the validation cohort 1; (**C**) the AJCC tumor staging in the validation cohort 2; (**D**) the new risk classification in the training cohort; (**E**) the new risk classification in the validation cohort 1; and (**F**) the new risk classification in the validation cohort 2.

Using the nomogram and the findings from the X-tile software (Version 3.6.1), we classified the patients into the following three categories based on their total score: low risk (total score < 461), middle risk (461 ≤ total score < 519), and high risk (total score ≥ 519) (Figure 7). The prognostic capability of this novel classification system was significantly better than that of the traditional AJCC TNM staging system, as demonstrated by the Kaplan–Meier curves (Figure 8).

### 3.6. Constructing a Web-Based Survival Calculator

**Figure 9:**
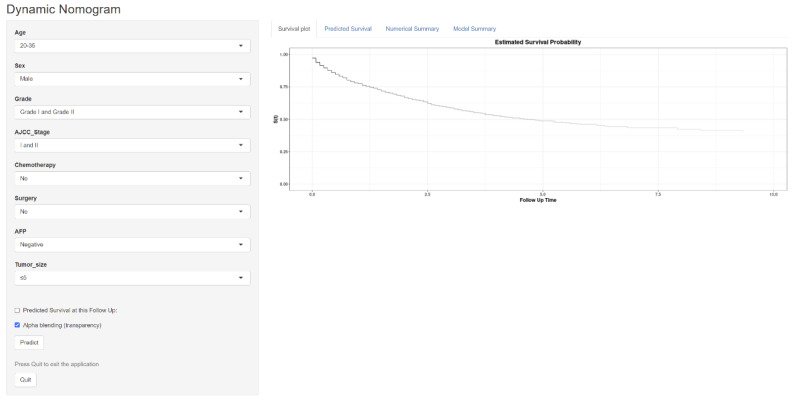
A web-based nomogram for predicting overall survival for HCC patients with EO-LIHC.

We have developed a publicly accessible web-based survival calculator (URL: https://doctoryyds.shinyapps.io/DynNomapp/ accessed on 2 November 2023) that utilizes the nomogram model to predict OS in EO-LIHC patients based on their personalized characteristics (Figure 9). The survival calculator collects variables, such as age, sex, AFP level, tumor grade, tumor stage, tumor size, surgery, and chemotherapy. For instance, a male patient aged 20–35 years with EO-LIHC, who had a Grade I or II tumor and underwent chemotherapy and surgery, would have a predicted 5-year OS rate of 60% (95% CI: 50–72%). The website is user-friendly and convenient and aims to assist in the individualized prediction of outcomes for EO-LIHC patients.

## 4. Discussion

Hepatocellular carcinoma has traditionally been more common among the elderly than among young patients [17]. However, recently, a noticeable increase in the incidence of EO-LIHC has been observed. The rise in early-onset cancers may be partly attributed to increased screening efforts and improved early-detection methods before the age of 50. The accurate prediction of a survival prognosis is crucial for healthcare professionals to facilitate individualized treatment and follow-up decisions. Although the AJCC TNM staging method is currently the most popular prognostic evaluation technique, focusing exclusively on the anatomical infiltration and metastasis of the tumor may restrict the accuracy of the survival prognosis [18,19,20]. Recently, several clinical prediction models that offer superior predictive power compared to the AJCC TNM staging system have emerged, expanding the options for the accurate prediction of tumor prognosis.

There are relatively few clinical prediction models for EO-LIHC patients. This study included 1392 EO-LIHC patients. Univariate and multivariate Cox regression analyses conducted to identify independent prognostic factors revealed nine such factors, including age, sex, tumor grade, surgery, chemotherapy, tumor size, and AFP level. Prognostic line graph models were constructed based on these factors. The performance of these models was evaluated using various assessment methods, including the C-index for a discriminant analysis, calibration curves for a calibration assessment, and the DCA for evaluating clinical usefulness. Additionally, a web-based OS prediction model specifically tailored for EO-LIHC patients was created and validated. Overall, these advancements contribute to improved decision-making and personalized care for individuals with EO-LIHC.

The AFP was initially identified as a tumor-associated antigen and a target for immunotherapy in HCC [21]. The AFP has been widely used for HCC surveillance and evaluating treatment response in HCC patients [22,23]. The results of this study corroborate the conclusions of previous studies that EO-LIHC patients may have increased AFP levels as a distinct risk factor. These results are also consistent with clinical observations and support the clinical significance of AFP as a prognostic marker in EO-LIHC.

Tumor size is a significant factor that influences the prognosis of hepatocellular carcinoma (HCC), and it is commonly used as a crucial criterion for HCC staging and treatment guidelines [24]. Several multicenter studies focusing on surgically-resected HCC have consistently demonstrated that tumors measuring >5 cm are associated with a poorer prognosis than those measuring ≤5 cm [25,26,27]. The nomogram clearly illustrates the relationship between the tumor size and prognosis, indicating that as the tumor size increases, the corresponding score also increases, indicating a worse prognosis. This observation underscores the importance of considering tumor size when assessing the prognosis of HCC patients.

The findings of this study also show that both chemotherapy and surgery function as distinct protective factors. Surgical resection remains the treatment of choice for HCC [28]. However, due to late-stage diagnosis and the lack of symptoms in early-stage HCC, many patients are not eligible for surgery [29]. Current guidelines may not endorse surgery for all cases of HCC [30,31]. However, a retrospective analysis by Mao et al. showed that patients who received viable surgery had better results than those who did not, even in the presence of distant metastases [32]. Additionally, a combination of surgery and chemotherapy has been shown to be beneficial for patients with HCC [33,34]. Systemic chemotherapy, utilizing agents such as gemcitabine, doxorubicin, or combination regimens, has shown improved survival rates in patients with HCC [33]. Consistent with these findings, the nomogram scores indicate that patients who received chemotherapy had a more favorable prognosis compared to those who did not. Beyond these mentioned factors, research has elucidated that cancer patients grappling with obesity exhibit a less favorable prognosis, potentially linked to the burdensome load of excessive body composition [35]. Conversely, individuals contending with underlying conditions like diabetes tend to experience a more promising prognosis [36]. These additional dimensions contribute to the intricate landscape of prognostication within the realm of cancer care, necessitating a holistic approach to comprehending and improving patient outcomes.

The nomogram is a simple-to-use statistical tool that considers various risk variables to offer patients personalized evaluations [37,38]. In this study, a new risk model was developed based on the risk points derived from the column line plot, allowing for the identification of high and low-risk patients with EO-LIHC [39]. The clinical relevance of this risk model was confirmed through an independent analysis, validating its predictive effectiveness across diverse patient populations. As the culmination of this research, we have developed the first online survival calculator for patients with EO-LIHC. This tool considers personalized clinicopathological features and can help predict postoperative outcomes on an individual basis.

This study has several limitations. First, it was a retrospective analysis, which introduces inherent limitations in data collection and potential bias. Second, data regarding important prognostic factors, such as etiology, hepatitis B surface antigen status, aspartate transferase levels, and vascular infiltration, were not available in the SEER database, which may have affected the comprehensive evaluation of prognostic factors. Third, the SEER database predominantly represents the US population; therefore, the findings may not be generalizable to other regions or populations. Fourth, although this study included a case-cohort from the Chinese population, the sample size was relatively small, which may limit the generalizability and overall robustness of the findings.

## 5. Conclusions

This study aimed to enhance the prediction of OS in EO-LIHC patients by combining data from the SEER database and a cohort from our hospital. Thus, a precise and specific nomogram was developed to predict OS in EO-LIHC patients. Moreover, a novel risk model and web-based survival calculator were developed for clinical application. These efforts intend to enhance the prognostic evaluation and individualized treatment decision-making in EO-LIHC patients.

## Figures and Tables

**Table 1:** Patients’ characteristics.

Characteristics	Training Cohort	Validation Cohort 1	*p* Value
n	899	388	
Age, n (%)			0.662
≥35	685 (53.2%)	300 (23.3%)	
<35	214 (16.6%)	88 (6.8%)	
Sex, n (%)			0.418
Male	693 (53.8%)	291 (22.6%)	
Female	206 (16%)	97 (7.5%)	
Race, n (%)			0.859
White	536 (41.6%)	225 (17.5%)	
Other	246 (19.1%)	111 (8.6%)	
Black	117 (9.1%)	52 (4%)	
Grade, n (%)			0.966
Well/Moderate	366 (28.4%)	158 (12.3%)	
Poor/Undifferentiated	158 (12.3%)	66 (5.1%)	
Unknown	375 (29.1%)	164 (12.7%)	
AJCC_Stage, n (%)			0.699
Unknown	166 (12.9%)	64 (5%)	
stage I–II	393 (30.5%)	174 (13.5%)	
stage III–IV	340 (26.4%)	150 (11.7%)	
Stage_T, n (%)			0.218
Unknown	179 (13.9%)	65 (5.1%)	
T1/T2	474 (36.8%)	201 (15.6%)	
T3/T4	246 (19.1%)	122 (9.5%)	
Stage_N, n (%)			0.163
Unknown	180 (14%)	63 (4.9%)	
N0	648 (50.3%)	286 (22.2%)	
N1	71 (5.5%)	39 (3%)	
Stage_M, n (%)			0.633
Unknown	73 (5.7%)	29 (2.3%)	
M0	656 (51%)	293 (22.8%)	
M1	170 (13.2%)	66 (5.1%)	
Dissected lymph nodes, n (%)			0.176
0	820 (63.7%)	344 (26.7%)	
1–3	69 (5.4%)	35 (2.7%)	
≥4	10 (0.8%)	9 (0.7%)	
Surgery, n (%)			0.765
No	564 (43.8%)	240 (18.6%)	
Yes	335 (26%)	148 (11.5%)	
Chemotherapy, n (%)			0.248
No	551 (42.8%)	251 (19.5%)	
Yes	348 (27%)	137 (10.6%)	
AFP, n (%)			0.101
Negative	178 (13.8%)	90 (7%)	
Positive	477 (37.1%)	213 (16.6%)	
Unknown	244 (19%)	85 (6.6%)	
Tumor_size, n (%)			0.358
≤5 cm	317 (24.6%)	135 (10.5%)	
5–10 cm	209 (16.2%)	94 (7.3%)	
>10 cm	173 (13.4%)	87 (6.8%)	
Unknown	200 (15.5%)	72 (5.6%)	
Tumor_Number, n (%)			0.133
1	876 (68.1%)	372 (28.9%)	
2	23 (1.8%)	16 (1.2%)	

**Table 2:** Univariate and multivariate analyses for OS.

Variable	Univariate		*p*	Multivariate		*p*
	HR	95% CI		HR	95% CI	
Age						
<35	Reference					
≥35	1.45	1.19–1.77	<0.001	1.68	1.36–2.09	<0.001
Sex						
Female	0.63	0.51–0.76	<0.001	0.67	0.54–0.82	<0.001
Male	Reference			Reference		
AJCC Stages						
I and II	Reference			Reference		
III and IV	4.11	3.41–4.95	<0.001	2.59	1.59–2.52	<0.001
Unknown	2.48	1.97–3.11	<0.001	1.81	0.99–1.49	<0.001
Grade						
Well	Reference			Reference		
Bad	2.68	2.15–3.34	<0.001	2	1.59–2.52	<0.001
Unknown	2.19	1.82–2.64	<0.001	1.21	0.99–1.49	0.067
Surgery						
No	Reference			Reference		
Yes	0.23	0.19–0.28	<0.001	0.3	0.24–0.38	<0.001
Chemotherapy						
No	Reference			Reference		
Yes	1.03	0.88–1.21	0.72	0.52	0.44–0.62	<0.001
AFP						
Negative	Reference			Reference		
Positive	2.71	2.14–3.43	<0.001	1.94	1.51–2.48	<0.001
Unknown	1.54	1.18–2.01	<0.01	0.91	0.67–1.22	0.52
Tumor size						
≤5 cm	Reference			Reference		
5–10 cm	2.22	1.79–2.76	<0.001	1.36	1.07–1.73	<0.05
>10 cm	2.32	1.84–2.92	<0.001	1.33	1.01–1.76	<0.05
Unknown	2.95	2.37–3.67	<0.001	1.27	0.96–1.69	0.099
Dissected lymph nodes						
0	Reference			Reference		
1–3	0.57	0.41–0.79	<0.001	0.89	0.63–1.25	0.499
≥4	0.26	0.08–0.81	<0.05	0.47	0.15–1.52	0.208

**Table 3:** NRI and IDI to evaluate the predictive power of the model.

Index	Training Cohort		*p*	Validation Cohort		*p*
	Value	95% CI		Value	95% CI	
NRI						
1-year OS	0.794	0.636–0.929		0.757	0.326–0.927	
3-year OS	0.699	0.211–0.814		0.521	0.292–0.783	
5-year OS	0.204	0.099–0.328		0.223	0.114–0.558	
IDI						
1-year OS	0.197	0.161–0.243	<0.001	0.228	0.161–0.300	<0.001
3-year OS	0.204	0.156–0.251	<0.001	0.306	0.224–0.398	<0.001
5-year OS	0.189	0.145–0.237	<0.001	0.323	0.234–0.406	<0.001

## Data Availability

The datasets used and analyzed during the current study are available from the corresponding author on reasonable request.

## Supplementary Materials

The following supporting information can be downloaded at: https://www.mdpi.com/article/10.3390/cancers15225310/s1, Table S1. Characteristics of patients in external validation cohort.
