# Supplementary material for: Construction of a Nomogram to Predict Overall Survival in Patients with Early-Onset Hepatocellular Carcinoma: A Retrospective Cohort Study
Source: Cancers (Basel). 2023 Nov 7;15(22):5310. doi: 10.3390/cancers15225310 (PMC10670167; doi:10.3390/cancers15225310)
Supplement: Supplementary file 1 [file cancers-15-05310-s001.zip › cancers-2696375-supplementary.pdf]

Table S1. Characteristics of patients in external validation cohort

**Characteristics**	**Validation cohort 2 (n=105)**
**Age, n (%)**	
≥40	79 (5.7%)
＜40	26 (1.9%)
**Sex, n (%)**	
Male	85 (6.1%)
Female	20 (1.4%)
**Marital_status, n (%)**	
Divorced	23 (1.7%)
Married	82 (5.9%)
Single	0 (0%)
Other	0 (0%)
**Race, n (%)**	
White	0 (0%)
Other	105 (7.5%)
Black	0 (0%)
**Grade, n (%)**	
Unknown	49 (3.5%)
Poor/Undifferentiated	26 (1.9%)
Well/Moderate	30 (2.2%)
**AJCC_Stage, n (%)**	
stage I-II	15 (1.1%)
Unknown	43 (3.1%)
stage III-IV	47 (3.4%)
Dissected lymph nodes, n (%)	
0	91 (6.5%)
1-3	11 (0.8%)
≥4	3 (0.2%)
**Surgery, n (%)**	
No	69 (5%)
Yes	36 (2.6%)
**Chemotherapy, n (%)**	

No	74 (5.3%)
Yes	31 (2.2%)
**AFP, n (%)**	
Negative	31 (2.2%)
Positive	74 (5.3%)
Unknown	0 (0%)
**Tumor_size, n (%)**	
≤5cm	23 (1.7%)
5-10cm	45 (3.2%)
＞10cm	37 (2.7%)
**Tumor_Number, n (%)**	
1	103 (7.4%)
2	2 (0.1%)
